# Enhancing the classification of spectrally similar land use/land cover classes using transfer learning in arid regions

**DOI:** 10.1038/s41598-026-38540-5

**Published:** 2026-02-26

**Authors:** Nourhan H. Farag, Dina Abdelhafiz, Mohamed A. Abdrabo, Mohamed A. ELIskandarani, Mahmoud A. Hassaan

**Affiliations:** 1 Environmental Studies Department, Institute of Graduate Studies and Research, Alexandria University, Alexandria, Egypt; 2 Informatics Research Institute, City of Scientific Research and Technological Applications (SRTA-City), New Borg El Arab, Egypt

**Keywords:** Land Use/Land cover (LULC) classification, Semantic segmentation, Bareland areas, Transfer learning, Resnet50-FPN model, Climate sciences, Ecology, Ecology, Environmental sciences, Mathematics and computing

## Abstract

Accurate mapping of Land Use/ Land Cover (LULC) in arid and semi-arid areas is essential for managing urban planning, monitoring environmental risks, and preserving the ecosystem balance. Conventional classification techniques provide inaccurate mapping of LULC in such areas due to the spectral similarity behavior of built-up and bareland classes. To address this challenge, semantic segmentation techniques have been recently employed to enhance classification accuracy. Meanwhile, in the case of imbalanced datasets, these models still exhibit limited generalization ability. To overcome this limitation, this study proposes a transfer learning approach to enhance the classification accuracy between built-up and bareland classes in the Nile Delta in Egypt, utilizing an imbalanced dataset. Four transfer learning models were employed, including Resnet50-Unet, Resnet50-FPN, Resnet50-PSPNet and Unet and their results were compared with the Maximum Likelihood classifier. The results showed that Resnet50-FPN achieved the highest F1-score of (0.877), followed by Resnet50-Unet (0.8705), Resnet50-PSPNet (0.852), Unet++ (0.792) and Maximum Likelihood (0.725). These findings highlight the effectiveness of transfer learning in enhancing LULC classification performance compared to the conventional Maximum Likelihood classifier. The superior performance of Resnet50-FPN is due to its multi-scale feature extraction capability, which enables more effective representation of LULC classes while preserving the spatial information of remotely sensed images. This indicates that the ResNet50-FPN classifier can be employed in different contexts to differentiate between built-up and bareland areas.

**Supplementary Information:**

The online version contains supplementary material available at 10.1038/s41598-026-38540-5.

## Introduction

 Generally, Earth’s surface is divided into land use and land cover surfaces. Land use refers to human activities on Earth’s land, such as industrial and urban development and agricultural lands. While land cover refers to the biophysical features on land, such as water, natural vegetation and soil. Dynamics of global LULC patterns are rapidly changing due to population growth, economic growth, and anthropogenic activities^1^. These changes generally have a multifaceted role, as changes of LULC result in urban planning, which is accompanied by negative implications on biodiversity, food security, and climate change^2,3^. Accordingly, understanding LULC dynamics is crucial in identifying the main drivers of such dynamics, followed by supporting decision-makers in monitoring and managing changes in LULC patterns, thereby preserving ecosystem resources (Sundar and Deka, 2021). This highlights the importance of modeling LULC dynamics, which requires accurate LULC mapping, which is usually retrieved through the classification of remotely sensed data.

Usually, data of LULC can be retrieved through multi-spectral and hyperspectral satellite images, which provide spectral and spatial information. There are different launched satellite sensors with varied spatial resolution of remotely sensed data representing LULC patterns. Accordingly, sensors can be categorized into high and medium spatial resolution. Quickbird, Ikonos-2, satellite sensors have high spatial resolutions of 0.61 m and 0.82 m, respectively. While Sentinel-2, Landsat 7 (ETM+), Landsat 8 (OLI), and Landsat 9 (OLI) of medium spatial resolution, ranging between 10 and 30 m. Geospatial information extracted from remotely sensed images is commonly analyzed using Geographic Information Systems^4^.

It is worth noting that the classification of remotely sensed data to retrieve LULC patterns in arid and semi-arid regions, which are characterized by highly diverse landforms, is challenging in terms of distinguishing between built-up and bareland areas due to their similar spectral signature. Several studies assessed the performance of different remote sensing and Machine Learning (ML) techniques to provide more accurate mapping of LULC classes. Meanwhile, it was argued that these techniques are considered inappropriate to be applied in arid and semi-arid regions to differentiate between built-up and bareland areas. To address this issue, Deep Learning (DL) techniques were proposed to benefit from the spatial and spectral information of remotely sensed data, and revealed higher performance than conventional techniques, especially in complex patterns of LULC classes^5–7^.

However, most of the studies applying DL in LULC classification focused mainly on hyperspectral images, with limited applications in multispectral images^8^. In addition, these studies concentrated on high spatial resolution with few applications on medium (10–30 m) spatial resolution data, despite their availability on Landsat and Sentinel-2 imageries. This is because high spatial resolution data contain more detailed features that assist in empowering the performance of the classification process through DL models^9^. This gap can be addressed by employing transfer learning models using the freely available multispectral imagery then the fine-tuning approach can be achieved to increase the DL accuracy to differentiate between built-up and bareland classes that have similar spectral signatures.

### Related work

Common conventional remote sensing classification techniques are applied to delineate LULC classes from remotely sensed data, such as Maximum Likelihood, Minimum Distance and Spectral Indices techniques. The Maximum Likelihood and Minimum Distance, which are calculated based on the mean value of the predefined classes^10,11^, showed poor performance in the case of overlapped classes^12^, and the existence of outliers^11^. Accordingly, differentiating between built-up and bareland areas in arid and semi-arid regions is challenging using Maximum Likelihood Minimum Distance and statistical-based techniques. To overcome this limitation, the spectral resolution of remotely sensed data is utilized for mapping LULC using the spectral indices technique^13–17^. Despite the systematic approach of Spectral Indices in delineating LULC classes, there is misclassification between built-up and bareland areas in arid and semi-arid regions, where bareland areas have a flat topography structure and drought soil, constraining proper classification, especially in dry climate^18^. With the advances in satellite technology, the availability of remotely sensed data allows for employing ML techniques in the remote sensing field.

Accordingly, various ML techniques have been employed to improve the differentiation between built-up and bareland classes due to their generalization capability^19^, employing different techniques for obtaining massive training datasets^20^. For instance, Random Forest, combined with distinct class factors, has been used to enhance classification accuracy. This is to benefit from the characteristics of ensemble learning to capture detailed texture information of classes through the intrinsic importance ranking^19,21,22^. ML techniques show high classification performance in distinguishing built-up and bareland areas in humid conditions such as tropical regions, where bareland regions are characterized either by moist soil or dense vegetation cover^23^. Arguably, conventional remote sensing classification techniques have poor performance in differentiating between built-up and barren land in arid and semi-arid regions due to their focus on the spectral level in data processing and classification.

Recently, DL classification techniques have been applied to process semantic features of remote sensing images by applying feature engineering transactions, taking into account the spectral and spatial resolutions of LULC classes^24,25^. In this respect, semantic segmentation techniques revealed superior performance over conventional classification techniques in complex patterns of LULC classes as well as classes with similar spectral behavior in accordance with the scalability of datasets and spatial resolution of training samples^6,7^. In the case of imbalanced datasets, semantic segmentation models resulted in a relatively low accuracy^26^. Regarding spatial resolution of the training samples, it was argued that most of the semantic segmentation models were trained using high spatial resolution, approximately less than 10 m, which may not be freely available with a big dataset^9,27–29^, and limited studies focusing on medium spatial resolution images. For empowering semantic segmentation techniques in the case of a limited dataset, the transfer learning approach is applied to improve the classification performance of LULC classes. This is due to its capability to improve the model generalization and achieve better performance in efficient computation time^30–32^.

### Egypt: LULC classification techniques

The Nile Delta in Egypt is a vital area, accounting for approximately 63% of the cultivated land in the country^33^. Meanwhile, due to anthropogenic activities, the Nile Delta has witnessed the urban encroachment issue, which led to the importance of accurate mapping of LULC for monitoring changes in LULC patterns^34,35^.

**Fig. 1:**
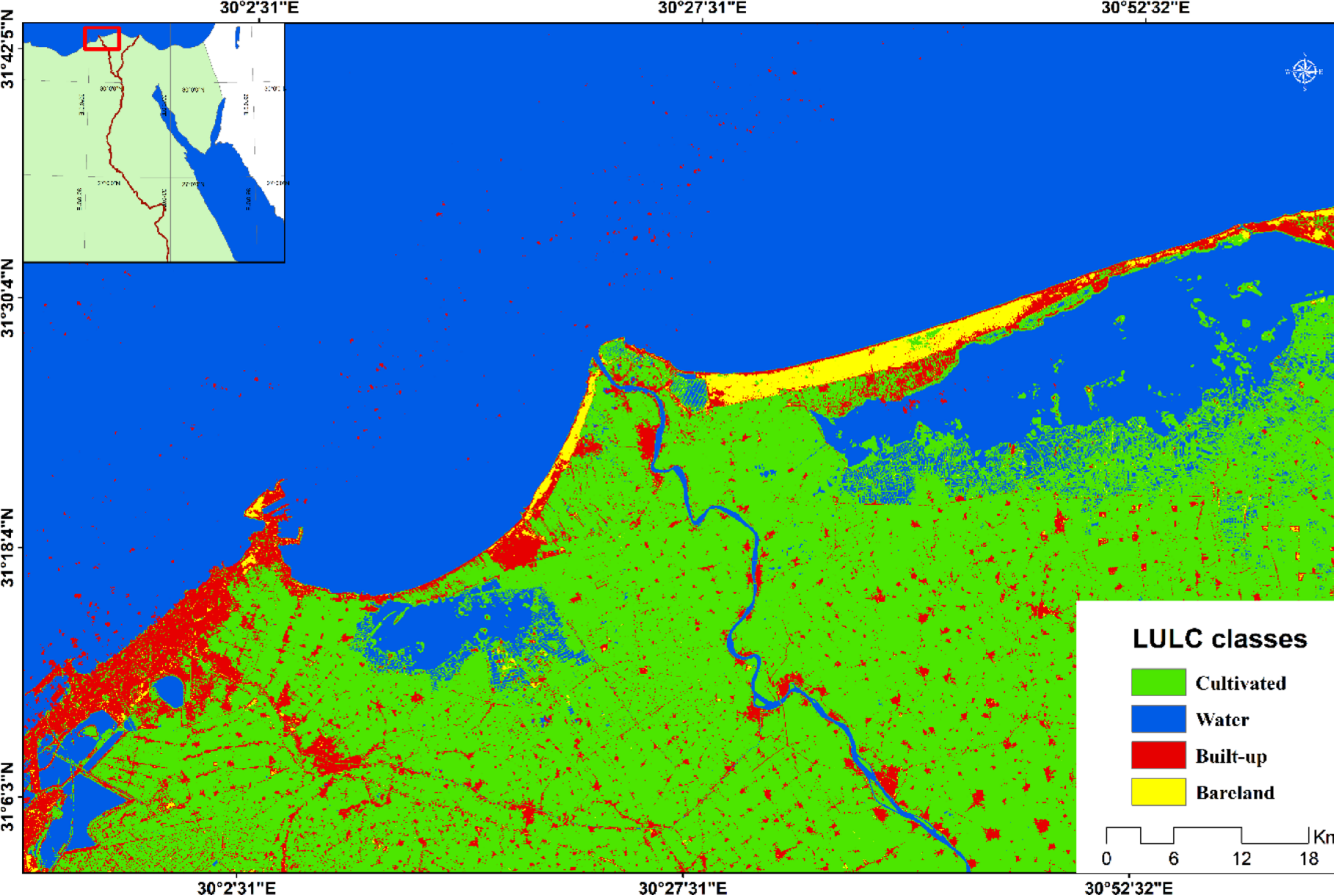
The Northern strip of the Nile Delta in 2022 (map created using ARCGIS Desktop ver. 10.8).

The Northern strip of the Nile Delta, which comprises the coastal zone of the Nile Delta extending for about 250 km from Alexandria in the West to Burullus Lake in the East, is selected as the study area (Fig. 1)^36^. This region has a complex LULC pattern, including bareland, built-up area, wetlands and cultivated land. In such a context, it is difficult to distinguish between bareland and built-up areas from satellite images through conventional classification techniques^37,38^. It has been argued that semantic segmentation techniques have not yet been implemented in such a study area to classify LULC and differentiate between built-up and bareland areas. Accordingly, it is essential to enhance the classification of LULC in the Northern strip of the Nile Delta and improve the differentiation between built-up and bareland by applying the transfer learning approach.

The main objective of this article is to employ transfer learning models to enhance the classification accuracy between built-up and bareland classes in the case of the Nile Delta in Egypt, utilizing an imbalanced dataset.

The outcome of this research can assist in:

- Enhancing the accuracy of mapping complex and overlapping LULC classes, as well as classes with similar spectral signatures in arid and semi-arid regions, using DL techniques.
- Utilizing open-source, medium spatial resolution satellite images of LULC in the case of an imbalanced dataset for classification using transfer learning techniques. This was achieved first by training the transfer learning models on the prepared ground truth dataset of medium spatial resolution of LULC classes, which captures the general features of these classes. This was followed by fine-tuning the trained models using the imbalanced datasets.

## Methodology

**Fig. 2:**
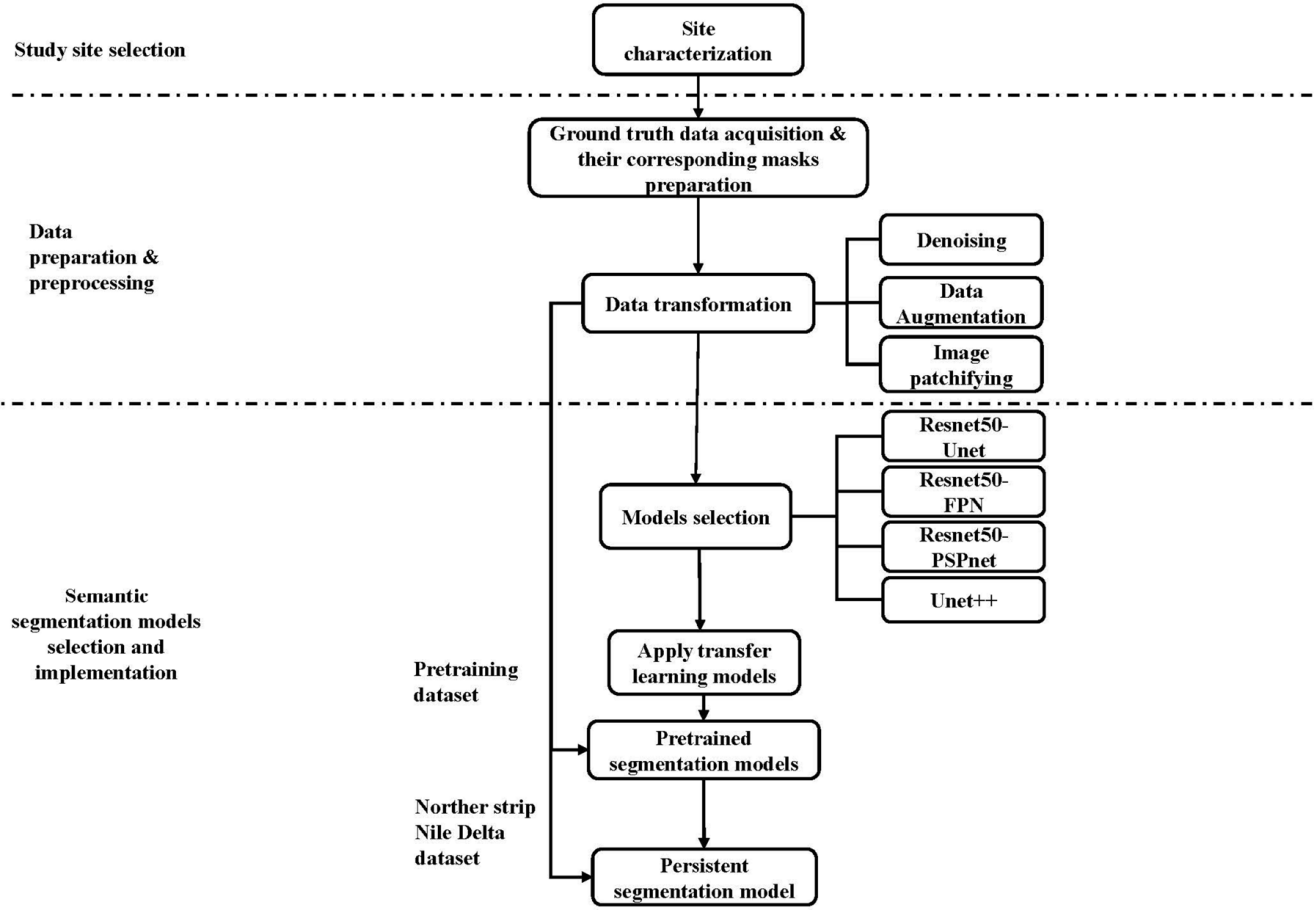
Proposed methodology of developing LULC segmentation model.

Enhancing the classification between built-up and bareland classes using the transfer learning approach, entails applying various semantic segmentation models, assessing their performance, and selecting the most appropriate one. For this purpose, a two-step methodology was suggested (Fig. 2), preparing and preprocessing the relevant data, and developing a transfer learning LULC segmentation model.

### Data Preparation and preprocessing

The dataset of the Northern strip of the Nile Delta was found to be imbalanced, which tends to poor generalization performance and biased results. Accordingly, another balanced dataset representing the South-western side of the Nile Delta, pretraining dataset (Figure S1) was prepared to ensure balanced LULC classes. This allows the pretrained models to learn and extract the general features of LULC. Thereafter, the pretrained models are fine-tuned using the imbalanced data^30^.

The dataset, including satellite images of LULC classes and their thematic maps, is usually acquired through multiple sources, such as well-known benchmark datasets^39^, the USGS Earth Explorer portal^40,41^, and the Google Earth Engine (GEE) platform^6^. However, benchmark datasets are not available in developing countries, and the USGS Earth Explorer portal provides only satellite images, so their thematic maps are required to be prepared by experts. Meanwhile, GEE provides satellite images and thematic maps of LULC classes with clear cloud cover. For our case study in Egypt, GEE was utilized for acquiring and processing multispectral medium spatial resolution satellite images to produce thematic maps depicting LULC patterns in subsequent points of time, utilizing the Random Forest algorithm, applying the same methodology discussed in^42^. This involves acquiring the satellite images representing the study area and manually labeling pixels of each LULC class by experts in the remote sensing field. The samples were then prepared to train the RF classifier. Finally, the trained model generates classified LULC thematic maps for satellite images. Classified thematic maps were validated by experts in the Remote Sensing field in the Environmental Studies department.

The prepared dataset was divided into two groups: the pretraining dataset, which involves 104 images (Table 1) and the Northern strip of the Nile Delta dataset, including 79 images (Table 2), targeting learning more specific features of the LULC to allow for enhancing the classification between built-up and bareland areas. The total number of dataset is 183 images of Landsat 8 Operational Land Imager and Thermal Infrared Sensor (OLI/TIRS) Level-1 were used in this study. The data of Green, Red, NIR and SWIR bands were processed as top-of-atmosphere (TOA) reflectance with a spatial resolution of 30 m and acquired for the period 2013–2022. To ensure data quality, scenes were filtered by cloud cover percentage, and the least cloudy images were selected using the JavaScript function *sort(‘CLOUD_COVER’).*

The two dataset groups were preprocessed as follows:

- Minority class weighting: the two datasets were found to be imbalanced. For the dataset, the majority class was cultivated, followed by bareland, built-up and the minority water class. For the Northern Strip of Nile Delta dataset, water was found to be the majority class, followed by cultivated, bareland and the minority class was found to be the built-up class (Table 3). Accordingly, the class weighting function “***compute_class_weight****()*” was employed for both datasets during the training and validation process to assign higher weights for minority classes.
- Noise Removal: pixel values with zero and negative values are replaced with a marginal value of $\:{10}^{-6}$ to prevent division issues of data normalization.
- Feature normalization: satellite images are normalized using the Min-Max scaling function.
- Data augmentation: typical data augmentation is applied, such as flipping, rotating, adding noise, or adjusting colors to improve the model generalization and prevent overfitting^43^. Meanwhile, in this study, the training data is augmented by selecting different band combinations. This is to benefit from the informative content of available satellite image bands. The selected band combinations of the original training samples of the two datasets are Green, Red, NIR and are augmented by selecting band combinations of Red, NIR, and SWIR.
- Data patchifying: based on the literature, the training dataset of Unet, FPN, and Unet + + was patchified into 256 × 256 × 3 dimensions^31^ to be ready for the convolution process. While the training dataset of the PSP-Net model was patchified to be 384 × 384 × 3 dimensions^44^.
- Data splitting: The dataset was randomly split into training and validation sets with a 90:10 ratio.

**Table 1:** Description of the pretraining dataset.

Model data split	Resnet50-Unet	Resnet50-FPN	Resnet50-PSP-Net	Unet++
Training and validation images	741	741	741	5738
Testing images	59	59	59	59

**Table 2:** Description of the Northern strip of nile delta dataset.

Model data split	Resnet50-Unet	Resnet50-FPN	Resnet50-PSP-Net	Unet++
Training and validation images	355	355	194	413
Testing images	20	20	20	20

**Table 3:** Number of pixels per class in each dataset.

Dataset	Cultivated	Water	Built-up	Bareland
Pretraining dataset	17,885,867	1,404,778	3,619,357	25,652,174
The Northern Strip of Nile Delta dataset	7,744,507	9,485,079	2,697,866	3,337,828

### Semantic segmentation models selection and implementation

To enhance the LULC classification, particularly the differentiation between built-up and bareland classes, four segmentation models were employed, including Unet, Feature Pyramid Network (FPN) and Pyramid Scene Parsing Network (PSPNet) as well and Unet++. Unet, either in its standard structure or modified versions, is frequently applied for LULC classification using Sentinel-1 and Sentinel-2^6,31,32^. FPN and PSPNet are pyramid-like structure models that apply feature extraction of objects at varying scales^45,46^, with limited studies evaluating their performance in differentiating between built-up and bareland classes in arid areas. Unet + + revealed high segmentation accuracy for complex patterns of objects^47^. To benefit from the transfer learning approach^48^, Unet, FPN and PSPNet were employed as pretrained models with Resnet50 as a backbone due to its structure of skip connections for extracting features of objects at varying scales^32^.

To improve LULC classification using an imbalanced dataset, the transfer learning approach was applied. The selected segmentation models were first trained using a balanced dataset of the South-western side of the Nile Delta. The output pretrained models were then fine-tuned using the imbalanced data of the Northern strip of the Nile Delta dataset. The pretrained model was trained using various combinations of batch sizes and epochs, ultimately determining optimal adjustment values according to the dataset and the High-Performance Computing (HPC) system capabilities (Table_S 1). The fine-tuned models were examined using different combination trials of batch size values, number of epochs, learning rate and optimizer to identify the appropriate values. It is worth mentioning that the same hyperparameter values were used for all models to ensure consistent comparison and to keep the computational cost manageable (Table 4).

**Table 4:** Hyperparameter configurations of the fine-tuned models.

Hyperparameter	Value
Batch size	8
Epochs	50
Learning rate	0.0001
Optimizer	Adam

The training, validation, and testing progress of the segmentation models was evaluated using the F1-score metrics and Intersection over Union (IoU). The F1-score is a comprehensive metric that measures the average score of Precision and Recall of the training model. As in some cases with similar spectral behavior of LULC classes, such as built-up and bareland classes, precision may be less informative, as it ignores false negative pixels and thus cannot predict actual pixels that are not recognized by the model. In contrast, Recall may be misleading as it ignores the false positive pixels, which may lead to a high Recall ratio. So, the F1-score balances both metrics and can be calculated by the following formula^49^:

1 $$\:{\boldsymbol{F}}_{1}=2\frac{\boldsymbol{T}\boldsymbol{P}}{2\boldsymbol{T}\boldsymbol{P}+\boldsymbol{F}\boldsymbol{N}+\boldsymbol{F}\boldsymbol{P}}$$

where.

**F**_1_ the F1-score value,

**TP**: the correctly classified pixel values in the positive class,

**FP**: the incorrectly classified pixel values in the positive class,

**FN**: the incorrectly classified pixel values in the negative class.

In addition, IoU can be used to evaluate the learning process of the segmentation models, as it measures the percentage of the overlapping area between the actual class and the predicted one through the following formula^49^:

2 $$\:\boldsymbol{I}\boldsymbol{o}\boldsymbol{U}=\:\frac{\boldsymbol{T}\boldsymbol{P}}{\boldsymbol{T}\boldsymbol{P}+\boldsymbol{F}\boldsymbol{P}+\boldsymbol{F}\boldsymbol{N}}$$

where.

**IoU**: the Intersection over Union,

**TP**: the correctly classified pixel values in the positive class,

**FP**: the incorrectly classified pixel values in the positive class,

**FN**: the incorrectly classified pixel values in the negative class.

The performance of the models was evaluated using a loss function, which was computed by integrating the Focal loss and Dice loss functions. Focal loss is estimated using the following formula to focus on the hard class to be recognized by the model, and emphasize the contribution of such a hard class to the total loss of the model^50^.

$$\user2{FL}_{{\left( {\user2{y},\user2{p}} \right)}} = - \left[ {\user2{\alpha }\;\user2{y}(1 - p)^{\user2{\gamma }} log(p) + (1 - \user2{\alpha })(1 - y)p^{\user2{\gamma }} log(1 - p)} \right]$$

where.

$\:{\boldsymbol{F}\boldsymbol{L}}_{\left(\boldsymbol{y},\boldsymbol{p}\right)}$the focal loss for a given actual label $\:y$and predicted value $\:p$,

**y**: the actual class value,

**p**: the predicted class value.

$\:\boldsymbol{\alpha\:}$ the weighting parameter for the class,

$\:\boldsymbol{\gamma\:}$the focusing parameter for hard classes.

If the value of $\:\boldsymbol{\gamma\:}$ → 0 means all classes are contributing equally to the model loss function, but if the value of $\:\boldsymbol{\gamma\:}$ > 0, the terms $\:{\left(1-\boldsymbol{p}\right)}^{\boldsymbol{\gamma\:}}$ and $\:{\boldsymbol{p}}^{\boldsymbol{\gamma\:}}$give the hard classes a higher contribution to the loss function of the model compared to easy classes.

Meanwhile, Dice loss is the complement of the Dice coefficient, which can be calculated using the following formula^50^.

3 $$\:\boldsymbol{D}=1-{\boldsymbol{D}}_{\boldsymbol{c}}$$

where.

**D**: the Dice loss function,

$\:{\boldsymbol{D}}_{\boldsymbol{c}}$ the Dice coefficient.

The Dice coefficient is calculated by the following formula:

4 $$\user2{Dice~}\left( {\user2{A},\user2{B}} \right) = \user2{~}\frac{{2\user2{~}\left| {\user2{A~} \cap \user2{~B}} \right|}}{{\left| \user2{A} \right| + \left| \user2{B} \right|}}$$

where,

**A**: the actual class,

**B**: the predicted class,

$\left| {\user2{A} \cap \user2{~B}} \right|$ the intersection between the actual and predicted class,

$\:|\boldsymbol{A}\cup\:\boldsymbol{B}|$ the union of actual and predicted class.

The experimental work was conducted using high-performance computing (HPC) with of Intel Xeon Gold 6248 processor with a 2.5 GHz clock speed, 128 GB of memory and of Tesla V100 GPU with 32 GB RAM. The implementation was applied using the Python programming language, with TensorFlow 2.10.1.

## Results and discussion

This section presents the performance of the pretrained models, using the pretraining dataset, as well as the fine-tuned models using the Northern Strip of Nile Delta dataset, highlighting the most appropriate model in differentiating between built-up and bareland in an arid region.

### Performance of LULC pretrained models

The IoU metric indicates that the pretrained models have a uniform performance during the training and validation process, as in Figure S2. The only exception was represented in the Resnet50-PSPNet, which showed a fluctuating pattern during validation progress. Such a fluctuation pattern may be attributed to the model structure, which is designed for recognizing the global context of objects; thus, it focuses more on recognizing the boundary of each LULC class than differentiating between the overlapped classes. Therefore, the Resnet50-PSPNet had the lowest validation IoU percentage (0.638) compared to Resnet50-Unet, Resnet50-FPN, and Unet + + that recorded 0.705, 0.720, and 0.738, respectively. Such results of IoU were found to be consistent with the reported results of applying semantic segmentation in classifying LULC^24^.

**Fig. 3:**
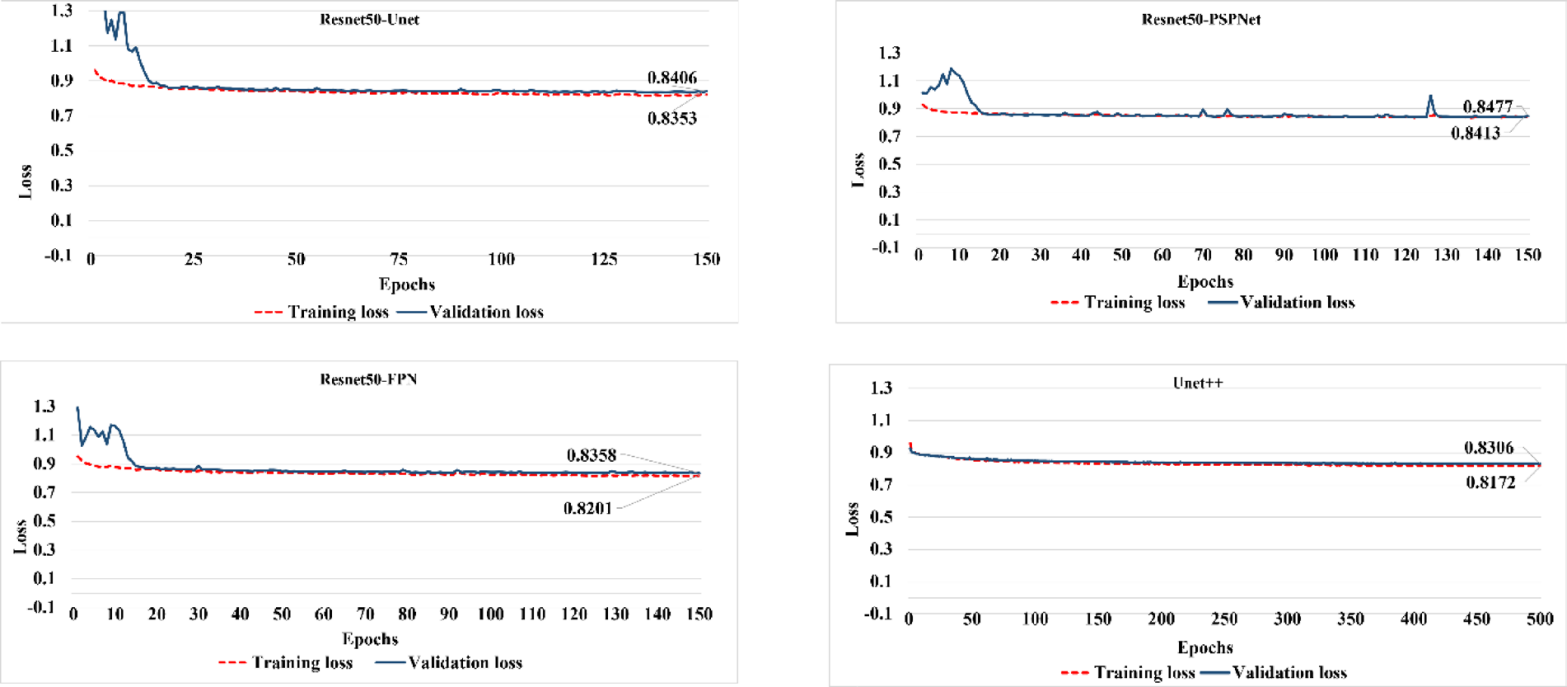
Loss function performance of the pretrained models.

The fluctuated pattern of the Resnet50-PSPNet model was highlighted by the results of the F1-score, which show that Resnet50-Unet, Resnet50-FPN, and Unet + + have more uniform performances compared to Resnet50-PSPNet (Figure S3), where the Unet + + and Resnet50-PSPNet recorded the highest (0.829) and lowest (0.746) F1-score values, respectively. The F1-score results confirm the findings from the IoU and loss function (Fig. 3), indicating that despite the overlapping patterns in LULC classes, the pretrained models are effectively learning the LULC features.

Compared to the training and validation process, the testing results revealed that the pretrained models have relatively less varied F1-score and IoU, particularly in the case of Resnet50-PSPNet, which achieved comparable performance to both Resnet50-Unet, Resnet50-FPN (Table 5). This was highlighted in the 2nd scene of the predicted image, where the Resnet50-PSPNet, compared to its masked image, clearly identified the buildings in the 6th of October city (Figure S4). While Resnet50-FPN and Resnet50-Unet models outperformed the masked images in identifying the green areas in the surrounding buildings, this highlights that the developed models outperformed ML techniques for LULC classification. The pretrained models are promising to be fine-tuned using the imbalanced dataset.

**Table 5:** Average F1-score and IoU index for testing the pretrained models.

Model	Average F1-score	Average IoU
Resnet50-Unet	0.850	0.772
Resnet50-FPN	0.842	0.763
Resnet50-PSPNet	0.858	0.768
Unet++	0.849	0.768

### Performance of LULC fine-tuned models

**Fig. 4:**
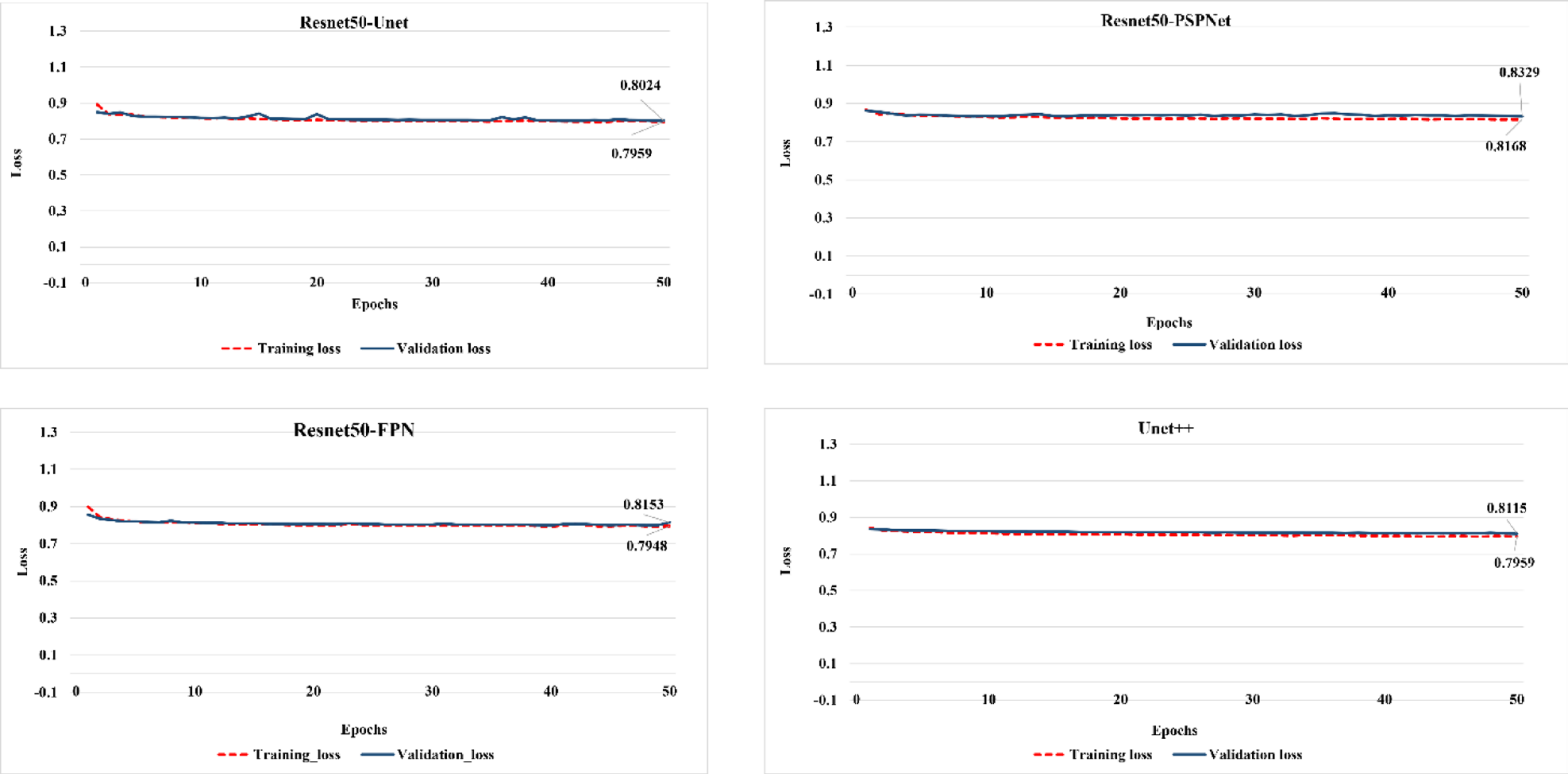
Loss function performance of the fine-tuned models.

It is worth noting that the loss function values for all fine-tuned models, except for Resnet50-PSPNet, improved by an average of 2% compared to the pretrained models, indicating that the fine-tuned models effectively learned the LULC class features. The results of loss function were 0.8024 for Resnet50-Unet, 0.8153 for Resnet50-FPN, 0.8329 for Resnet50-PSPNet, and 0.8115 for Unet + + with Resnet50-PSPNet having the lowest value (Fig. 4).

**Fig. 5:**
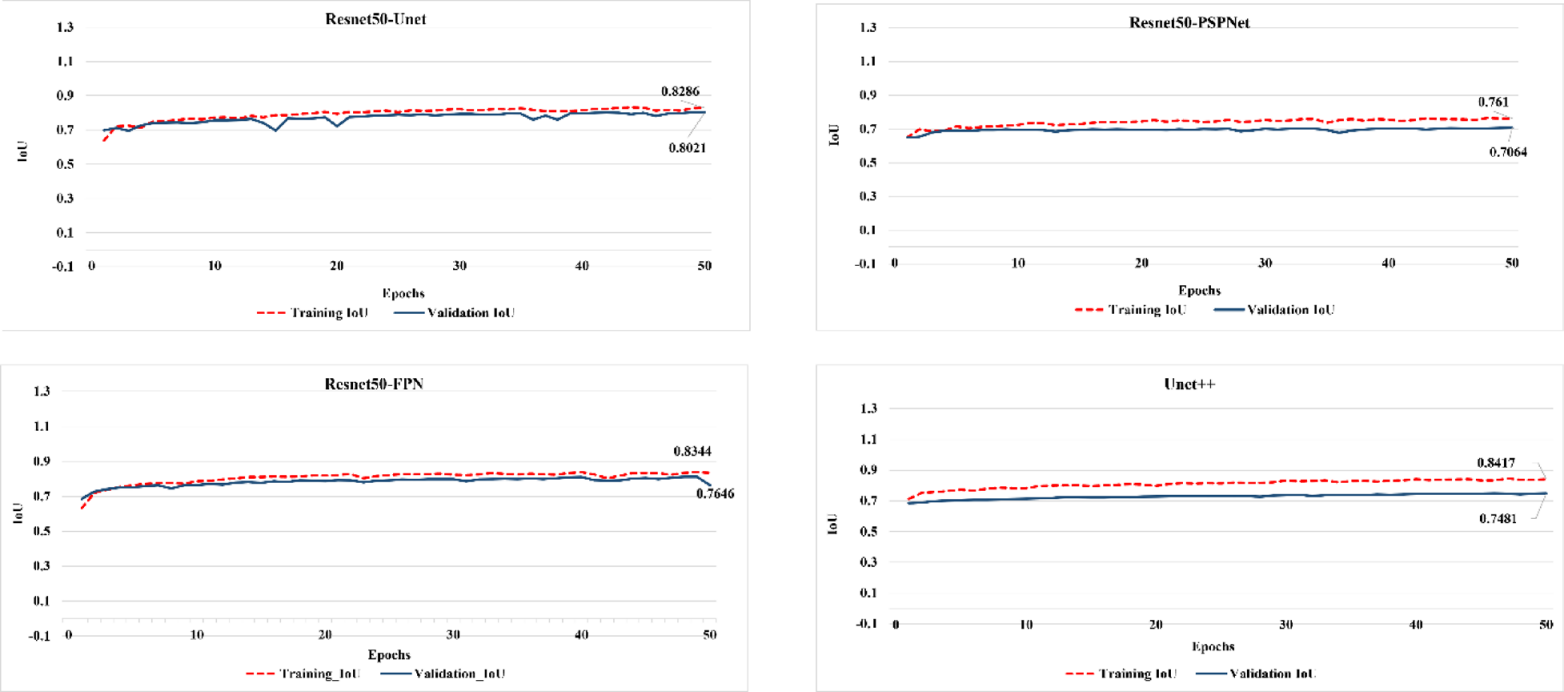
IoU performance of the fine-tuned models.

**Fig. 6:**
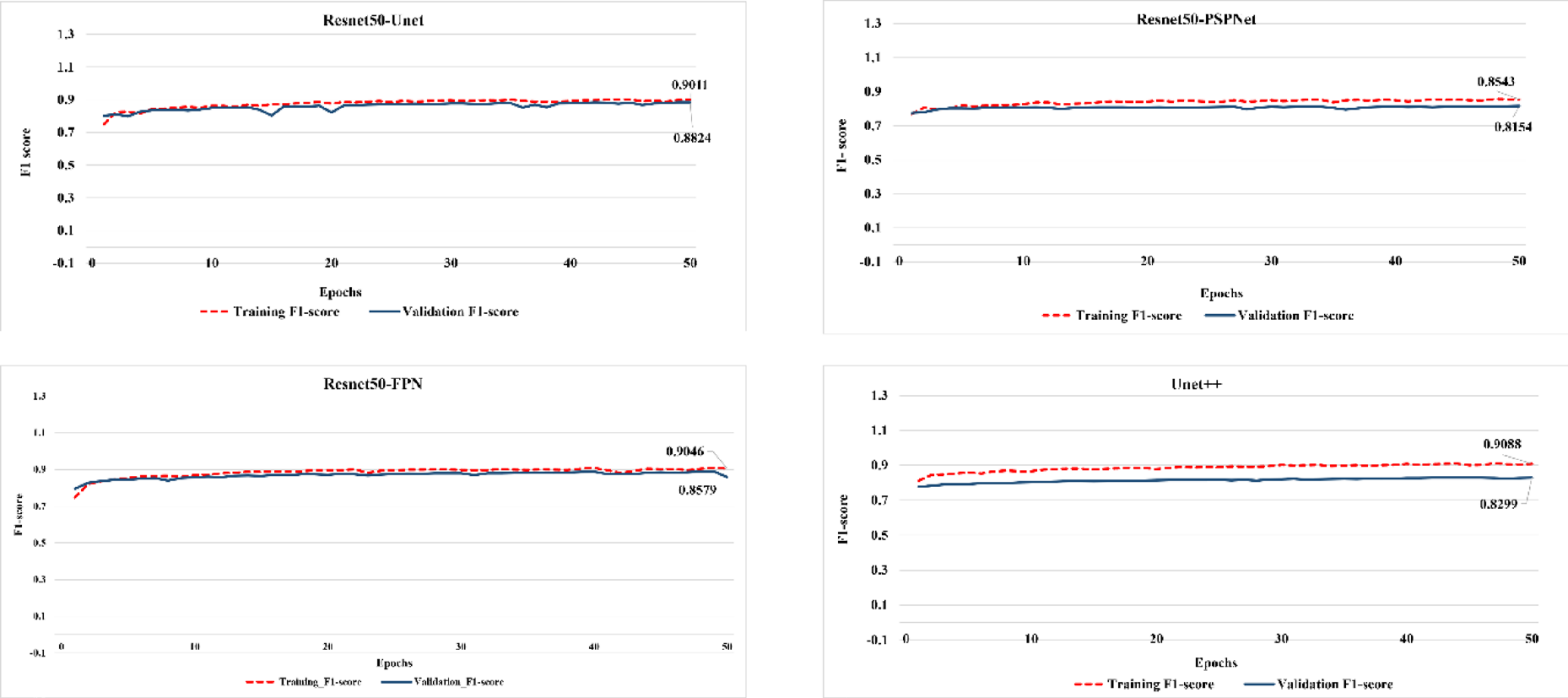
F1-score performance of the fine-tuned models.

Similar to the pretrained models, the fine-tuned Resnet50-Unet achieved the highest validation IoU score, while Resnet50-PSPNet revealed the lowest score (0.748) of IoU, while Resnet50-Unet recorded the highest score (0.802) (Fig. 5). The models were further evaluated using the F1-score, indicating consistency with the IoU results (Fig. 6), where all the fine-tuned models have an initial F1-score value of more than 0.7. This indicates that the models, in contrast to the pretrained models, have good start information regarding features of LULC classes, which was enhanced during the training progress. Generally, it can be argued that Resnet50-PSPNet has limited capacities for capturing the LULC features in the arid and semi-arid regions. Although the Resnet50-Unet model achieved the highest IoU and F1-score, the Resnet50-FPN model exhibited more steady training and validation progress for both metrics. This suggests that Resnet50-FPN effectively identified and learned the complex and overlapped features of classes through its pooling pyramid layers.

**Fig. 7:**
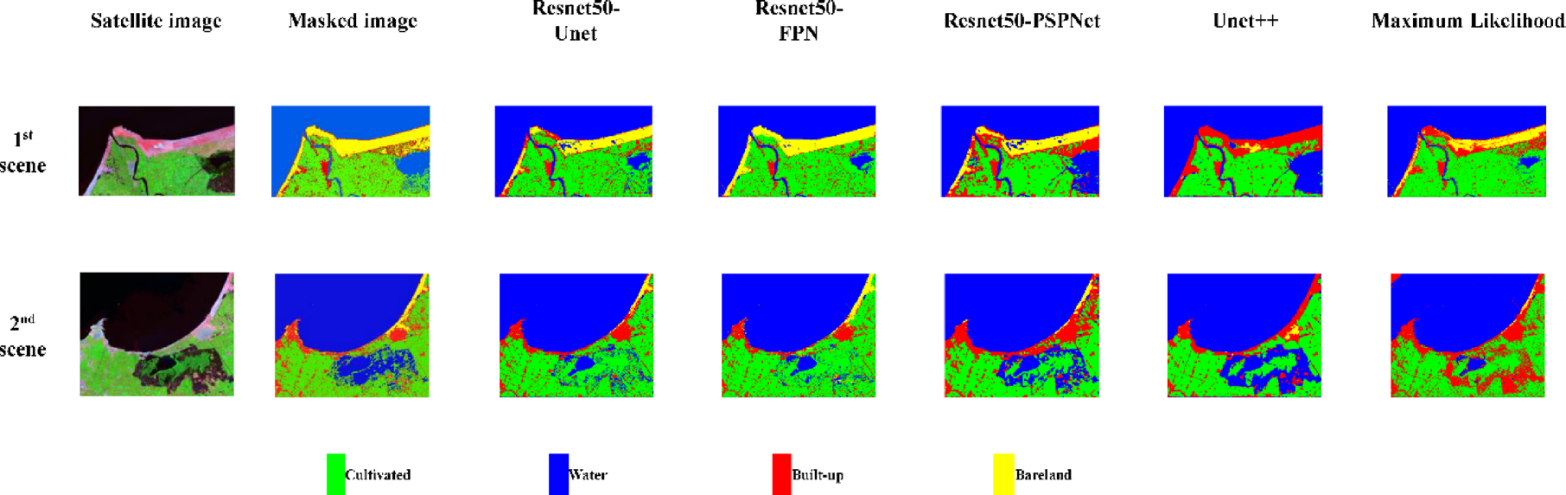
Predicted LULC classes of Northern strip of the Nile Delta using fine-tuned models compared to the Maximum Likelihood technique.

The fine-tuned models were applied to unseen data, and their performance was evaluated compared to the Maximum Likelihood technique, which is the most common conventional classification technique. It was found that the Maximum Likelihood technique has the lowest performance compared to the fine-tuned models (Table 6). Usually, Maximum Likelihood depends only on the pixel value range during classification, which results in misclassification of built-up and bareland classes that have similar spectral response and thus close pixel values. Resnet50-PSPNet concentrates more on identifying the general features of objects, that’s why it may misclassify overlapped LULC patterns. Despite that the Unet + + model has an efficient architecture in capturing low-level as well as high-level features through dense convolution blocks with skip connections, it showed the worst prediction results due to using relatively insufficient training samples. Meanwhile, Resnet50-Unet has limitations in capturing objects at small levels due to losing detailed features during the transformation of feature maps from encoder to decoder. Accordingly, a modified architecture of the Unet was suggested to enhance the segmentation of small objects^51^. ResNet50-FPN achieved nearly the highest performance in both F1-score and IoU, confirming its consistent progress during training and validation processes. In this respect, the predicted images of the fine-tuned models demonstrated that Resnet50-FPN surpassing the conventional LULC classification techniques as well as the fine-tuned segmentation models in enhancing the classification between built-up and bareland areas in the Northern strip of the Nile Delta (Fig. 7).

By comparing the results of the proposed approach in differentiating between built-up and bareland with the classification accuracy of another study that applied a semi-automated method in the same area, it was found that the developed segmentation models outperformed the semi-automated method in extracting barren land from built-up areas^38^. Also, the classification accuracy of the transfer learning approach is comparable to the studies that employed DL or ensemble learning of ML to LULC classification in arid and semi-arid countries^52,53^.

**Table 6:** Average F1-score and IoU of the fine-tuned models on testing scenes.

Model	Average F1-score	Average IoU
Resnet50-Unet	0.871	0.782
Resnet50-FPN	0.877	0.792
Resnet50-PSPNet	0.852	0.758
Unet++	0.792	0.705
Maximum Likelihood	0.725	0.604

For execution training and validation time, the Resnet50-Unet and Resnet50-FPN models recorded comparable execution time. Only 2 min of training and validation were taken by Resnet50-PSPNet, which can be attributed to its simple architecture compared to the other applied models (Table 7). By evaluating the testing complexity of the applied models, it was found that Resnet50-PSPNet had the shortest average inference time, Resnet50-Unet and Resnet50-FPN had similar average time, and Unet + + recorded the longest inference time due to its deep structure. By comparing the Maximum Likelihood technique to the applied transfer learning models, it was found that such technique took the longest time, almost two hours, to classify each image (Table 8). Generally, it was concluded that similar inference time was taken for the transfer learning models and did not exceed a fraction of a second, which is acceptable in remote sensing applications that span from seconds to several minutes.

**Table 7:** Training and validation execution time of the fine-tuned models.

Model	Inference time (Minute)
Resnet50-Unet	5
Resnet50-FPN	5
Resnet50-PSPNet	5
Unet++	7

**Table 8:** Average inference time for testing.

Model	Average inference time
Resnet50-Unet	11.5 (sec)
Resnet50-FPN	12 (sec)
Resnet50-PSPNet	9.5 (sec)
Unet++	17 (sec)
Maximum Likelihood	2 (hrs)

## Conclusion

Applying the transfer learning approach employing Resnet50-Unet, Resnet50-FPN, Resnet50-PSPNet, and Unet + + achieved effective learning of LULC features and resulting in enhanced classification between built-up and bareland areas in an arid region, where built-up and bareland classes have similar spectral signatures. While applying the Maximum Likelihood technique, a conventional technique, to acquire thematic maps of LULC in the Northern Strip of the Nile Delta in Egypt, revealed misclassification between built-up and bareland areas. This means that DL classification techniques give an enhanced classification accuracy for complex overlapping LULC classes due to the deep and robust structure for extracting features of LULC classes at both pixel and spatial levels. Resnet50-FPN has a superior performance in classifying complex overlapping LULC patterns and differentiating between built-up and bareland areas, compared to the other employed models. This highlights that the pyramid pooling modules structure of FPN enables multi-scale feature extraction for improved LULC classification accuracy, and distinguishing classes with the same spectral behavior. In addition, the structure of Resnet50 with skip connections as a backbone preserves the fine details of objects and prevents the vanishing gradient issue as well. For imbalanced datasets and medium spatial resolution LULC images, initially training the pretrained models on a balanced dataset followed by fine-tuning with the imbalanced dataset led to improved LULC classification performance. Meanwhile, this study encountered the limitation of using limited datasets in training fine-tuned models. To address this challenge, data from multiple satellite platforms (e.g., Landsat and Sentinel) should be combined under varying conditions, for complementary information, such as different seasons or cloud cover levels, to ensure consistency and enhance the generality of the analysis. For further enhancing the differentiation between LULC classes of similar spectral behavior performance, future efforts should focus on employing various transformers in integration with attention modules to give attention to the training model to recognize the fine detailed features of LULC classes in arid and semi-arid regions using medium spectral signatures.

## Supplementary Information

Below is the link to the electronic supplementary material.

Supplementary Material 1

## Data Availability

The datasets generated during the current study are available in the ***GitHub*** repository, ***https://github.com/nourhanhamdyebrahim123/LULC-classification/tree/main***.
