# Supplementary material for: Enhancing the classification of spectrally similar land use/land cover classes using transfer learning in arid regions
Source: Sci Rep. 2026 Feb 26;16:7729. doi: 10.1038/s41598-026-38540-5 (PMC12949008; doi:10.1038/s41598-026-38540-5)
Supplement: Supplementary file 1 — Supplementary Material 1 [file 41598_2026_38540_MOESM1_ESM.docx]

**Supplementary Information**

**Enhancing the Classification of Spectrally Similar Land Use/Land Cover Classes Using Transfer Learning in Arid Regions**

| 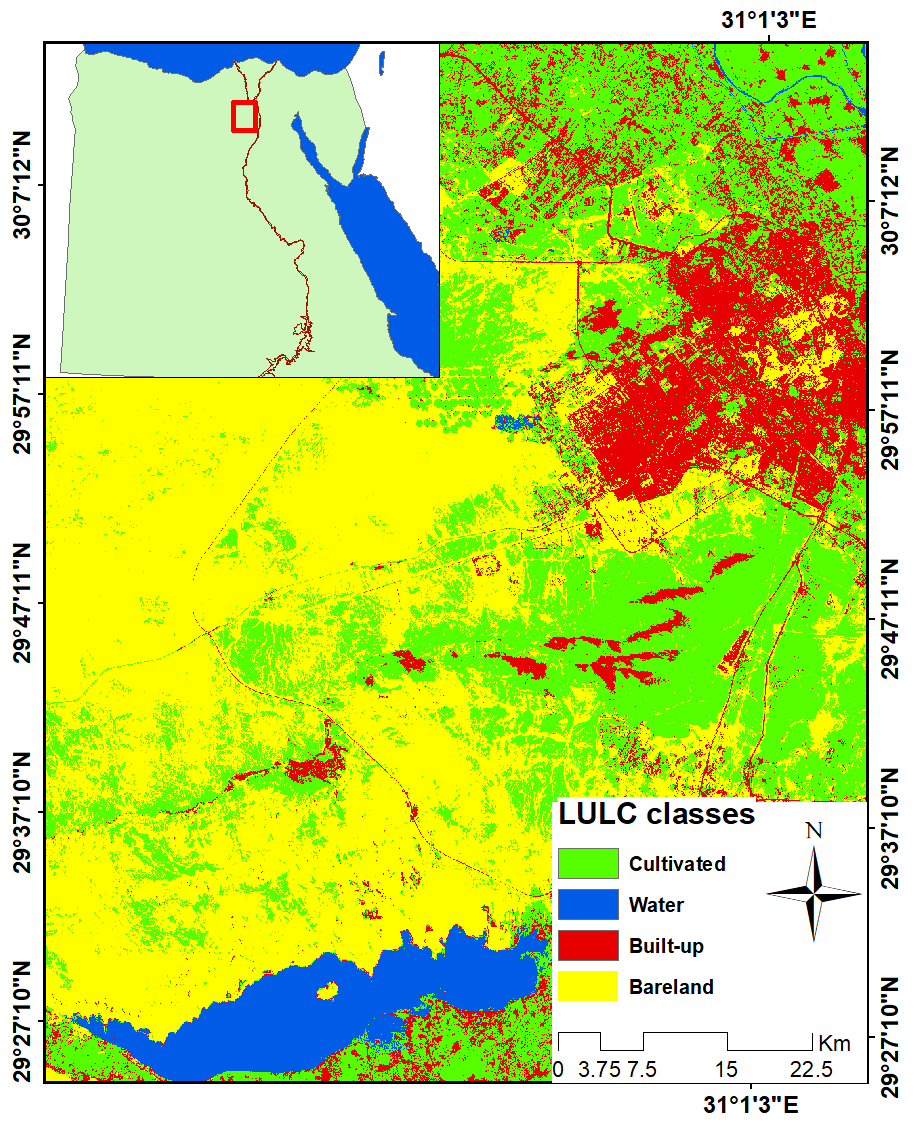 |
| --- |
| **Figure_S 1: The South-western side of the Nile Delta in 2022 (map created using ARCGIS Desktop ver. 10.8)** |

| **Table_S 1: Hyperparameters values of the segmentation models** | | |
| --- | --- | --- |
| **Hyperparameter** | **Model** | **Value** |
| **Batch size** | (Resnet50-Unet, Resnet50-FPN, Resnet50-PSPNet, Unet++) | 8 |
| **Epochs** | (Resnet50-Unet, Resnet50-FPN, Resnet50-PSPNet) | 150 |
|  | Unet++ | 500 |
| **Learning rate** | (Resnet50-Unet, Resnet50-FPN, Resnet50-PSPNet, Unet++) | 0.0001 |
| **Optimizer** | (Resnet50-Unet, Resnet50-FPN, Resnet50-PSPNet, Unet++) | Adam |

| **Resnet50-Unet** |
| --- |
| **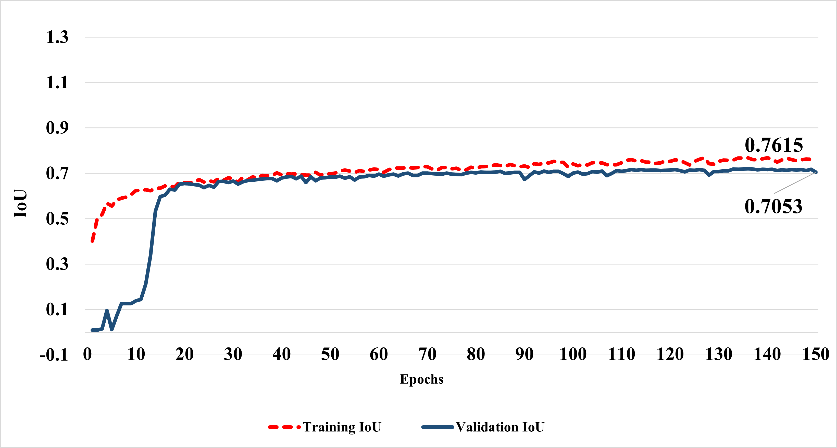** |
| **Resnet50-FPN** |
| **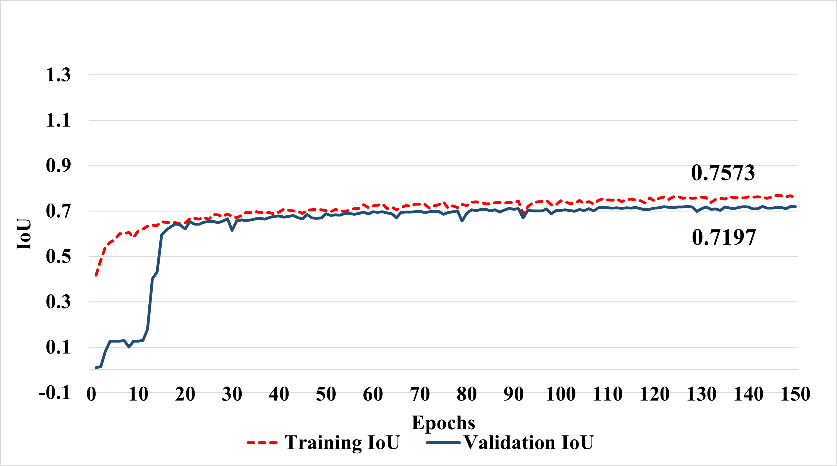** |
| **Resnet50-PSPNet** |
| **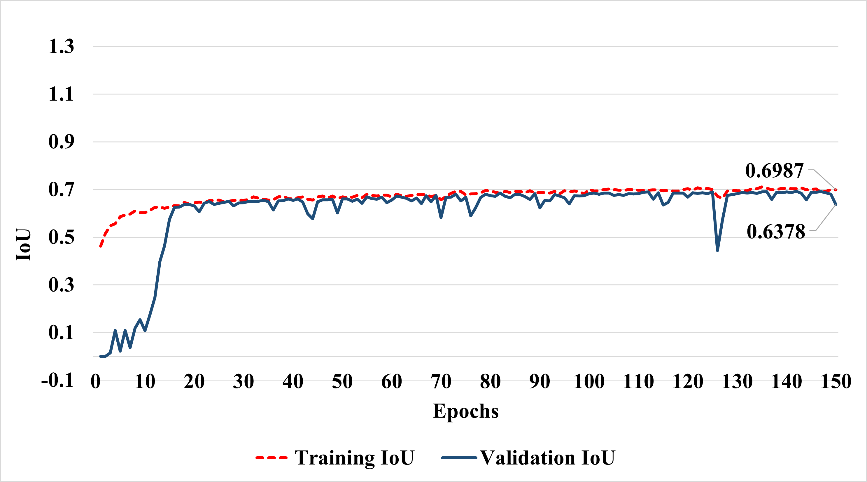** |
| **Unet++** |
| 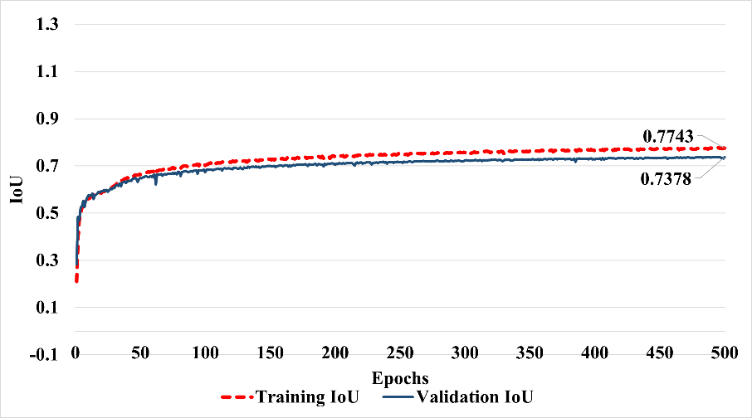 |
| **Figure_S 2: IoU performance of the pretrained models.** |

| **Resnet50-Unet** |
| --- |
| **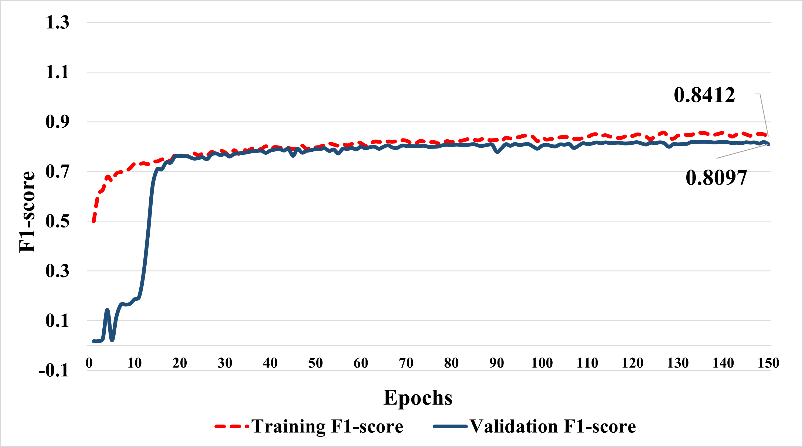** |
| **Resnet50-FPN** |
| 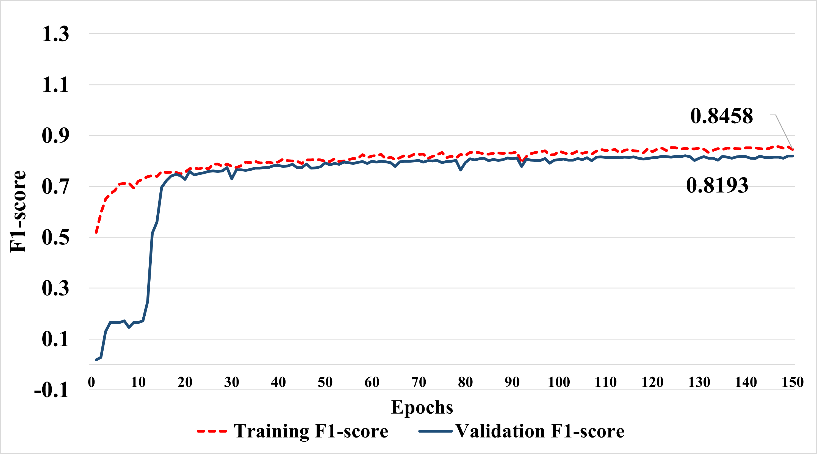 |
| **Resnet50-PSPNet** |
| **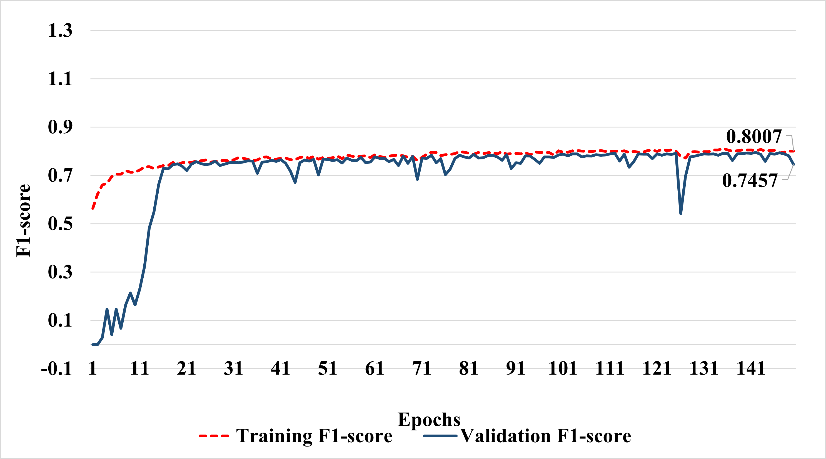** |
| **Unet++** |
| 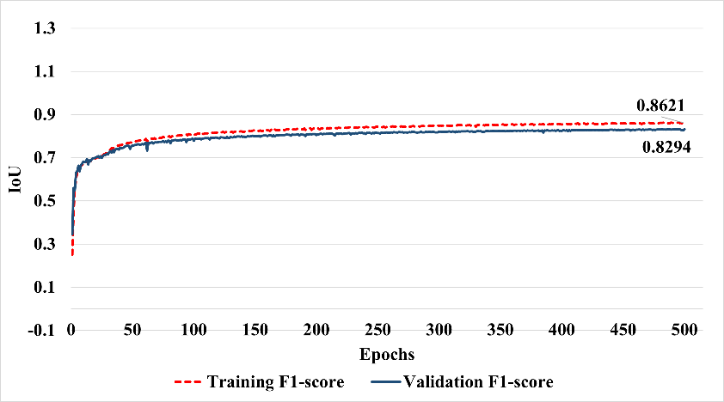 |
| **Figure_S 3: F1-score performance of the pretrained models** |

|  | 1^st^ Scene | | |
| --- | --- | --- | --- |
| Resnet50-Unet   \| Unet++ \| 1^st^ Scene \| \| \| \| --- \| --- \| --- \| --- \| \| 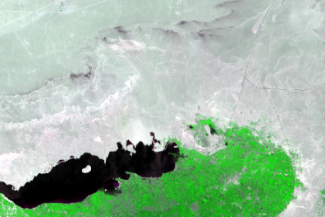 \| 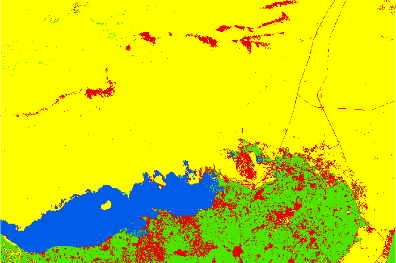 \| 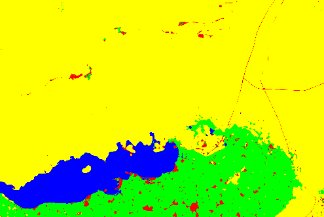 \| \| 2^nd^ Scene \| \| \| \| 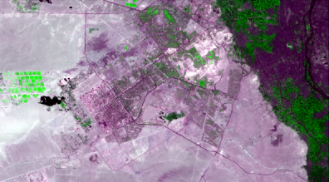 \| 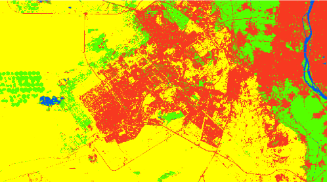 \| 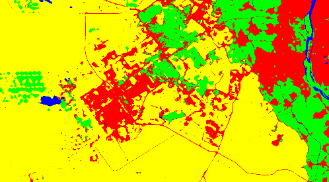 \| \| **Figure 1: Testing results of the pretrained models** \| \| \| \| | Satellite image | Ground truth image | Predicted image |
|  | 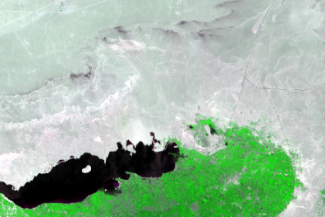 | 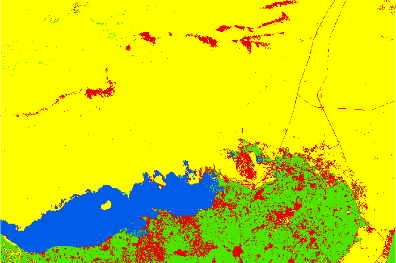 | 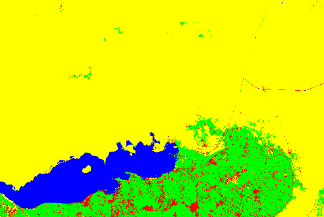 |
|  | 2^nd^ Scene | | |
|  | 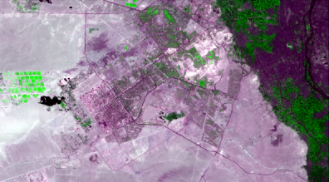 | 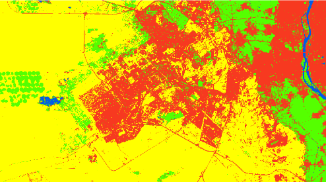 | 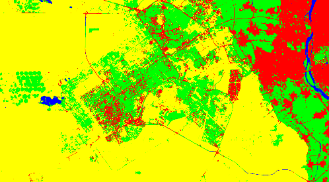 |
| Resnet50-FPN |  | 1^st^ Scene |  |
|  | 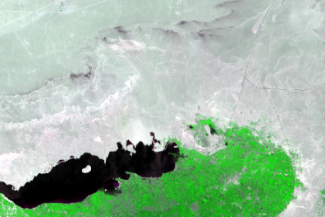 | 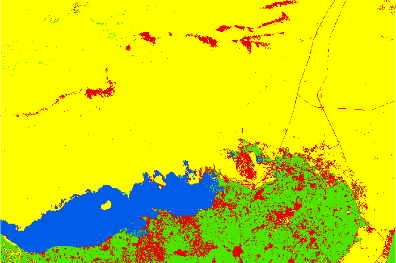 | 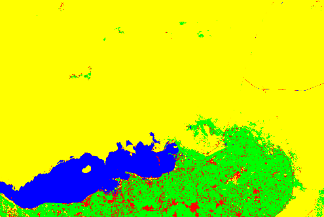 |
|  |  | 2^nd^ Scene |  |
|  | 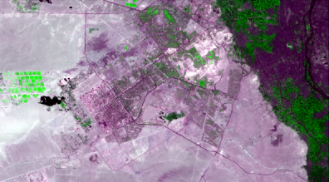 | 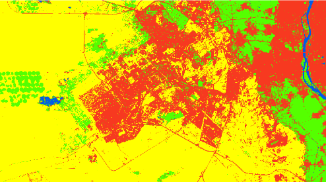 | 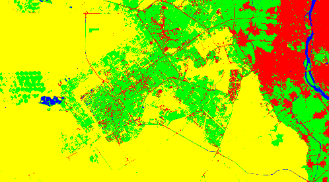 |
| Resnet50-PSPNet |  | 1^st^ Scene |  |
|  | 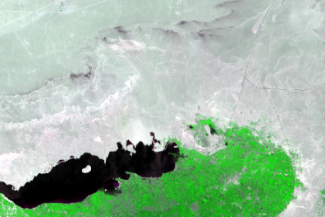 | 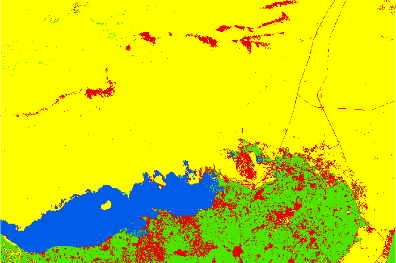 | 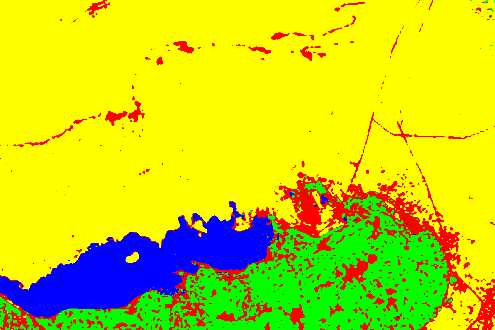 |
|  |  | 2^nd^ Scene |  |
|  | 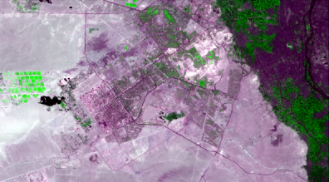 | 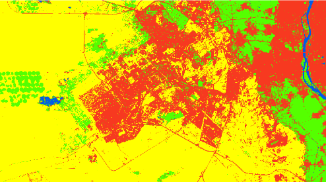 | 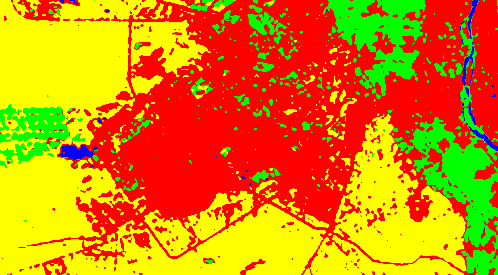 |
| Unet++ | 1^st^ Scene | | |
|  | 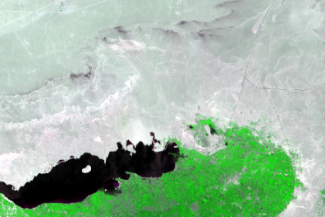 | 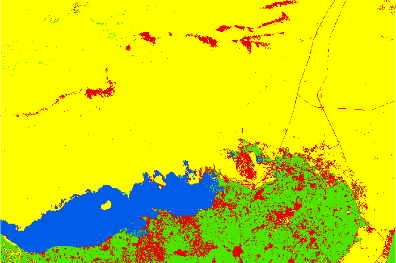 | 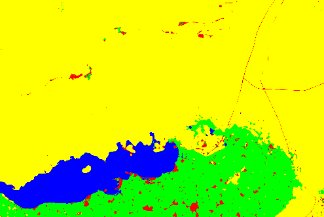 |
|  | 2^nd^ Scene | | |
|  | 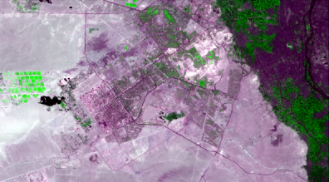 | 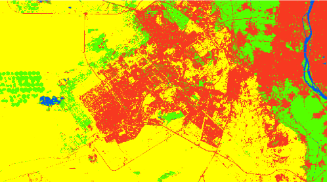 | 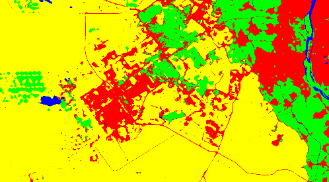 |
|  | **Figure_S 4: Testing results of the pretrained models. Satellite images and Masked images were generated using Google Earth Engine Platform (**[**https://earthengine.google.com/**](https://earthengine.google.com/)**). The predicted images were produced by the proposed DL model** | | |
